# Vestibular asymmetry in caloric and video head impulse testing: Do we interpret it correctly?

**DOI:** 10.1177/09574271251336143

**Published:** 2025-04-23

**Authors:** Maja Striteska, David Wexler, Ondrej Tichacek, Alfarghal Mohamad, Martin Chovanec, Erich Schneider

**Affiliations:** 1 Department of Otorhinolaryngology and Head and Neck Surgery, University Hospital Hradec Kralove, Faculty of Medicine in Hradec Kralove, 60564 Charles University, Hradec Kralove, Czechia; 2 Department of Otorhinolaryngology, Third Faculty of Medicine, Charles University and University Hospital Kralovske Vinohrady, Prague, Czechia; 3 Department of Otolaryngology, UMASS Chan Medical School, Worcester, MA, USA; 4 Institute of Organic Chemistry and Biochemistry of the Czech Academy of Sciences, Prague, Czechia; 5 Otolaryngology-Head and Neck Surgery, King Abdulaziz Medical City-Jeddah, Saudi Arabia; 6 Institute of Medical Technology, Brandenburg University of Technology Cottbus— 38871 Senftenberg, Cottbus, Germany

**Keywords:** vHIT, caloric test, Jongkees’ formula, bilateral test, unilateral test, vestibulo-ocular reflex, VOR, video oculography, norm, nonlinearity, asymmetry index

## Abstract

Caloric and video head impulse tests (vHITs) are essential for vestibular diagnostics, both employing Jongkees’ formula (JF) to quantify asymmetry. JF calculates unilateral weakness (UW) by subtracting the weaker ear (WE) response from the stronger ear (SE) and using the sum of both responses as a reference. However, the result is unwieldy and may mislead clinicians if interpreted as an indication of how much weaker the response of the WE is compared to the contralateral SE as a percentage. Through mathematical analysis, we examined what question JF answers and explored, for each vestibular test, alternative asymmetry equations for a more meaningful assessment of vestibular asymmetry. JF has three key limitations. First, its nonlinear nature leads to an underestimation of paresis, particularly when the WE response is near 41% of SE, where the calculated UW is capped at 18%. Second, JF derives the asymmetry from a “symmetry point”, splitting the difference between both sides, with the average response in the middle, rather than directly quantifying UW as clinicians understand it. Instead, JF answers two separate questions: “How much is the WE response below the average” and “How much is the SE response above the average.” To address these issues, a linear paresis calculation using only the SE response as a reference was later introduced. However, this approach did not resolve JF’s third limitation: artificially inflated values and sensitivity to small variations in WE when both ears are affected. Unlike the caloric test, the vHIT already relies on head velocity as an absolute reference for gain calculation, eliminating the need for SE in asymmetry calculation. Employing an ideal gain of 1, asymmetry can be expressed as a simple side-to-side gain difference, preventing inflated results in bilateral deficits and easing clinical calculation.

## Introduction

**Figure 1.**
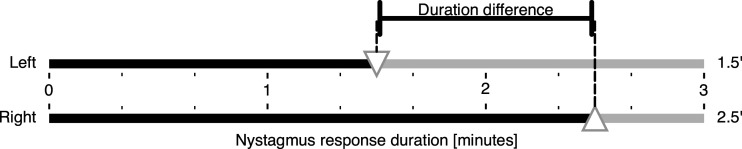
Nystagmus response durations and their difference resulting from monothermal caloric irrigation of the left (top) and right (bottom) ears. The metrics are visualized in a “calorigram” adapted from Fitzgerald and Hallpike.^2^ The reaction of the affected left ear in a hypothetical unilateral vestibular disorder is set to 60% of the stronger right ear.

Quantifying functional asymmetry between paired organs, such as the vestibular organs, is a common task in medical research, clinical practice and also sports science.^1^ In 1942, Fitzgerald and Hallpike calculated vestibular asymmetry from the oculomotor responses to caloric irrigation of the left and right ears—the caloric test—by the simple difference between nystagmus response durations expressed in seconds.^2^ Because duration differences were highly variable without standardization, in 1962, Jongkees expressed the left-right difference in excitability as a percentage of the total excitability of both ears,^3^ now known as the caloric asymmetry index. Later, Jongkees proposed the use of peak slow-phase velocity as the preferred excitability metric over nystagmus duration,^4^ and he also coined the term “calorigram” for the “convenient diagrammatic form” previously introduced by Fitzgerald and Hallpike for visualizing excitabilities and their differences (Figure 1).

In addition to the caloric test, Jongkees’ asymmetry index has more recently been applied also to the instrumental head impulse test.^5^ Both tests have been extensively investigated, for example, for their validity and test-retest reliability,^6,7^ and have become the cornerstones of vestibular diagnostics. Although the asymmetry index is also used in vestibular evoked myogenic potentials (VEMP),^8^ which have become part of the vestibular test battery, we will focus our discussion on the caloric test and the video head impulse test (vHIT) and begin our theoretical paper with a brief description of these two tests.

### Caloric test

The caloric test has been used for about a century to assess the integrity of the vestibular system; Barany’s research at the Vienna Clinic led to his Nobel Prize in 1914. It is a relatively simple test to perform, stimulating each side of the peripheral vestibular system separately. It primarily assesses the low frequency aspects of the two lateral semicircular canals. In the classic version of the test, each ear is stimulated by hot (H) and cold (C) water (44/30°C) or air (50/24°C) in four separate trials, allowing both the left (L) and the right (R) ears to be tested with both directions of the expected nystagmus response.

The primary clinical outcome of the caloric test is the unilateral weakness, which is also known as canal paresis or reduced vestibular response. This weakness is determined by comparing the responses of both ears and is expressed as a percentage of the unilateral weakness in the weaker ear. The four separate trials of the caloric test yield four nystagmus intensity metrics, which are used as the input variables to Jongkees’ formula (JF) to calculate the asymmetry index as its output,^3^ which is the measure of unilateral weakness:

(1) JF=((LC+LH)−(RC+RH))/(LC+LH+RC+RH)×100%

In addition, the directional preponderance (DP) reflecting the vestibular tone is calculated as the difference between the right and left beating nystagmus, expressed as a percentage of the total excitability:

(2) DP=((LC+RH)−(RC+LH)/(LC+LH+RC+RH)×100%

One limitation of the caloric test is that it does not provide an absolute reference value—a thermally induced endolymphatic flow velocity—to compare with the oculomotor response, making it difficult to establish a normal value for vestibular function. Another limitation is the possible presence of vestibular tone imbalance, reflected in a non-zero DP, which renders measurement of only one side—without comparison to the other—meaningless. Therefore, the caloric test is a comparative test that uses the response from both sides to determine the relative difference between them. This test assumes that one ear is normal or strong, whereas it is possible that both ears are deficient to varying degrees. Nevertheless, the test does help to determine if, and to what extent, one side is less responsive than the other.

### Video head impulse test

During impulsive testing in a healthy subject, the eye movement response will compensate for the head turn and the gaze will rest on the earth-fixed fixation target. This response of the vestibulo-ocular reflex (VOR) is typically evaluated based on its gain. Gain represents the output to input ratio of any dynamic system. To calculate the VOR gain, eye velocity or position is measured and compared to head velocity or position, respectively.

vHIT is a modern vestibular test that overcomes some of the limitations of the caloric test and provides important complementary information. It primarily assesses the high-frequency aspects of not only the two lateral semicircular canals but also of the four vertical semicircular canals. Unlike the caloric test, the vHIT provides a meaningful assessment of vestibular function even when performed on only one side, because it relies on an absolute reference value, head velocity, to calculate gain as the primary clinical outcome. This makes it possible to establish well-defined clinical limits and to define an ideal VOR gain of 1, reflecting concordant eye and head movements.

Although the value of calculating asymmetry in vHIT is questionable, as the reported gains already characterize vestibular function sufficiently by quantifying the function of each ear relative to the ideal gain, some studies and manufacturers have also adopted Jongkees’ formula to assess asymmetry in head impulse testing.^5,7^ In this case, the numerator consists of the difference between the two gains quantifying vestibular function for leftward (L) and rightward (R) head impulses, and the denominator is the sum of these metrics:

(3) JF=(L – R)/(L+R)×100%

In equations (1) and (3), we have presented Jongkees’ formula in its two variants for the caloric test and the vHIT, respectively. Since its introduction to the vestibular field in 1962, the formula has remained unchanged, despite the potential for misinterpretation. Clinicians may assume it directly represents, as a percentage, how much weaker the response of the affected (or weaker) ear is compared to the contralateral normal (or stronger) ear, which is not the case. While identifying vestibular asymmetry is undoubtedly important in clinical physiology, the definition and interpretation of left-right asymmetry have remained largely unchanged in clinical practice. This persists despite well-documented limitations and advancements in related fields, such as sports science.^1^ Here, we examine what question these traditional JF formulations actually answer and propose an alternative—a linear, more intuitive approach to expressing vestibular asymmetry. This refined method enhances the physiological understanding of vestibular test results and improves their clinical applicability.

## Asymmetry calculation

Calculating an asymmetry index (AI) is a common approach to quantifying left-right asymmetry but requires a reference value^9^ in the denominator to rescale the result to the interval between −100% and +100%, with the sign indicating the affected side. Without such standardization, the bare side-to-side difference proposed by Fitzgerald and Hallpike will remain unadjusted for and dependent on the scale of the variables being measured, and the limits will be indeterminate. A general formula for AI is:

$$\text{AI}=\left(\text{side}1-\text{side}2\right)/\left(\text{reference}\;\text{value}\right)\times 100\%$$

In JF, which we introduced in equation (1), the sum (total response) of four irrigations with hot and cold water of the left and right ear became the reference value in the denominator. In mathematics, such a reference value is known as the sum norm.^9^

### Unilateral and bilateral tests

While in the vestibular field the discussion on asymmetry calculation appears to be settled, as the formula has remained unchanged since Jongkees’ time, more recent research in sports science, for example, has uncovered conceptual challenges with a panoply of methods available for calculating the reference value.^9,10^ This value in the denominator should be chosen according to the test and its characteristics, as the choice of an inappropriate method may lead to results with both nonlinear and “artificially inflated” characteristics.^11^ In vestibular testing, however, it remains unclear which equation should be used to quantify the asymmetry between the two sides^12,13^ so that clinicians get the result they expect from the caloric test, and the same applies to vHIT.

In choosing the appropriate method, it is particularly important to distinguish between a unilateral test and a bilateral test.^12,13^ In the former case, the reference value should include information from only one side, whereas in the latter it should include information from both sides. The denominator has a major influence on the resulting asymmetry score^10^; the same difference between the responses of the two sides yields a lower asymmetry score when divided by the sum of the responses of both sides (bilateral test formula using the sum norm) than when divided by the response of the stronger side alone (unilateral test formula using the maximum or infinity norm^9^). This important distinction has not yet been made in the vestibular field.

The key assumption for using a bilateral formula such as JF is that both sides interact simultaneously during a task.^10^ While such a simultaneous irrigation of one ear with hot water and the other with cold water is conceivable in a scientific setting, the caloric test in its conventional Fitzgerald-Hallpike form, which is now widely used clinically, stimulates only one ear at a time and can therefore be considered a unilateral test. Nevertheless, the asymmetry between the two sides is still commonly calculated using JF,^3^ which takes the sum of both sides as the bilateral reference value, even though the use of a unilateral reference value would be more appropriate. VEMPs, another class of vestibular tests not discussed further, can be considered either unilateral, where stimuli are air-conducted sounds delivered separately to each ear, or bilateral, where bone-conducted sounds stimulate both ears simultaneously.^14^

For the vHIT, the same discussion arises as to whether it is a unilateral or bilateral test. Although the consequences of Ewald’s second law make the vHIT appear to be unilateral, it is still assumed that the contralateral side contributes to some extent because when the head is moved, both ears move together and the two vestibular organs interact as a push-pull pair with a gradual contralateral inhibition saturation, which is more evident in unilateral vestibular loss.^15–19^ This is especially true for low-velocity ipsilesionally directed head movements, as a residual contribution from the healthy contralateral side cannot be excluded.^20,21^ In contrast, “rapid […], unlike slow, […] head movements” are required to silence the contralateral input and ultimately “demonstrate […] asymmetry”^22^. In the former, low-velocity case, the test stimulus can be considered bilateral, whereas in the latter, high-velocity case—a mandatory prerequisite for proper head impulse testing—it is predominantly unilateral.

### The meaning behind Jongkees’ formula

We continue with a theoretical discussion of the question what JF actually answers. It appears that the answer is not directly related to the question typically asked by a clinician, who would intuitively expect JF to indicate the degree of response deficit in the weaker ear relative to the stronger ear. What it indicates instead can be seen by expanding both the numerator and the denominator in equation (4) by the same value of ½, a mathematical operation that doesn’t change the result of the fraction:

(4) JF=SE-WE/SE+WE×100%=12·SE-WE/12·SE+WE×100%

**Figure 2.**
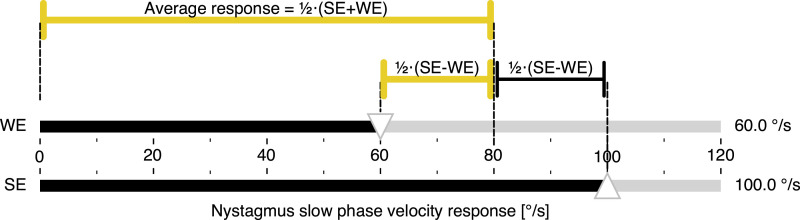
Calorigram of a hypothetical unilateral vestibular disorder illustrating the JF calculation. The nystagmus response of the affected WE (top) is set to 60% of the SE (bottom), corresponding to the clinical threshold asymmetry of 25% when calculated with JF. The magnitudes representing half the difference between the responses and the average response, corresponding to the numerator and denominator of JF, respectively, are color-coded as for JF in Figure 3 below. Abbreviations: Jongkees’ formula (JF), weaker ear (WE), stronger ear (SE).

The numerator is now half the side-to-side difference and the norm in the denominator is the average response, which is ½⋅(SE+WE). Therefore, JF calculates the percentage difference between each side and the average response by relating the difference to the average response. This is illustrated in Figure 2. More precisely, JF answers the two questions “how far is the weaker ear from the average (relative to the average)” and, at the same time, “how far is the stronger ear from the average (relative to the average),” as shown in a more rigorous mathematical derivation in the Appendix.

When the average response is considered a “symmetry point,” JF calculates how both sides simultaneously differ in percent from that point, which in the example in Figure 2 would be a nystagmus response of 80°/s. In other words, JF splits the difference between the two sides into two parts, with the average response in the middle. However, this “symmetry point” is not to be understood as an independent physiological operating point.

### Linearity and non-linearity

In 1994, Wexler^12^ was the first to point out the nonlinear nature of JF, which made the interpretation of its results unwieldy. As a solution, Wexler proposed to modify JF by replacing the total excitability or sum norm in the denominator with the maximum norm or excitability of the strong ear only, thus suggesting the use of a strong ear formula (SEF) as a means to linearize the asymmetry calculation. We performed a mathematical analysis to further investigate the nonlinearity of JF.

For proper mathematical analysis, it is convenient to adopt Wexler’s simplifications of JF by reducing the four-variable JF to a two-variable version using SE and WE for the total responses of the stronger and weaker ears, respectively, and then expressing the two-variable JF in terms of a dimensionless quantity, for example, a “weak ear relative to strong ear”: q = WE/SE. Assuming that the responses of both ears are positive, the range of q is the interval from 0 to 1. Then

(5) JF=(SE−WE)/(SE+WE)=(SE−SE·q)/(SE+SE·q)=(1−q)/(1+q)×100%

and similarly

(6) linear paresis (SEF)=(SE−WE) / SE=(SE−SE·q)/SE=(1−q)×100%

**Figure 3.**
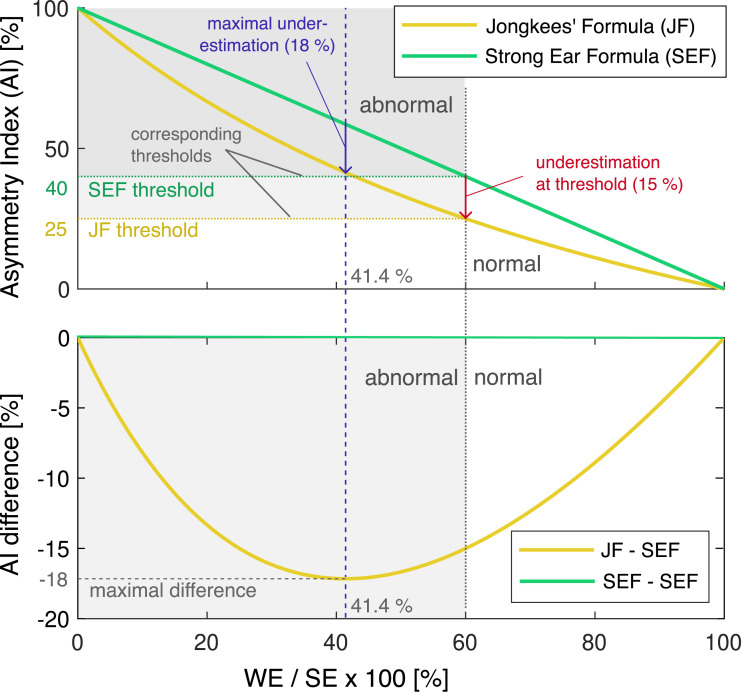
Comparison of asymmetry index calculations by linear SEF and nonlinear JF formulas (top) and their difference (bottom). When JF is used to estimate the unilateral weakness, its value is always underestimated, while SEF and unilateral weakness are identical. The normality threshold of JF = 25% corresponds to SEF = 40%. At this point, JF underestimates unilateral weakness by 15%. In the bottom plot, the JF - SEF difference acquires negative values (reflecting JF underestimation), reaching a maximum of −18% at 41.4% WE/SE. The top plot is adapted from Wexler.^9^ All graphs are plotted as a function of WE/SE in percent. Abbreviations: Jongkees’ formula (JF), weaker ear (WE), stronger ear (SE), strong ear formula (SEF), asymmetry index (AI).

Using equations (5) and (6), Figure 3 illustrates the nonlinear characteristic of JF compared to the linear SEF, as well as the difference between the two. JF always underestimates the paresis; the difference is greatest for q = $\sqrt{2}-1$ = 0.41, or in other words, when WE shows 41% of the SE response. At this point, the AI difference reaches a maximum of −18%. It is also noteworthy that the normality threshold of JF = 25% corresponds to SEF = 40%. At this point, JF underestimates the unilateral weakness by 15%.

**Figure 4.**
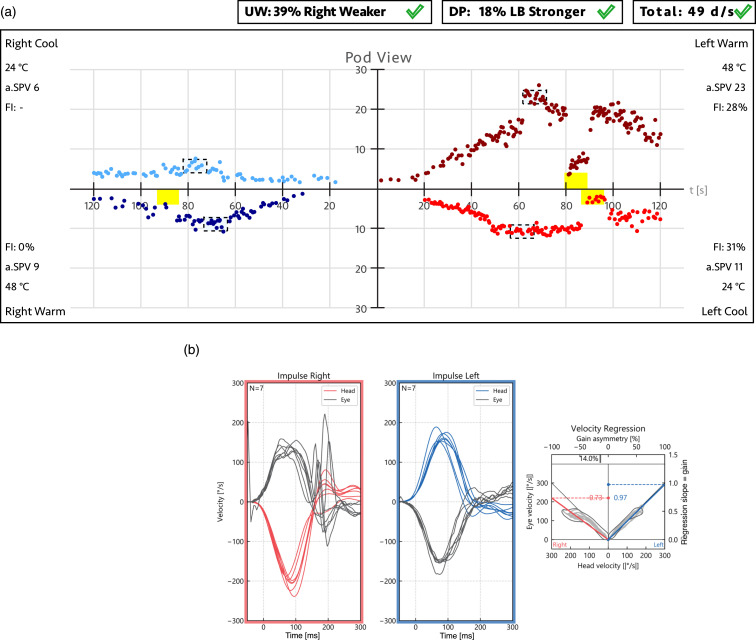
Typical caloric test result (a) from a patient with a unilateral vestibular deficit on the right side, confirmed by vHIT (b) Each quadrant in A represents the time course of the slow-phase velocity (SPV) response for a different caloric stimulation, with peak values occurring between 60 and 80 seconds. In this example, the peak SPV values are LH = 23°/s, LC = 24°/s, RH = 9°/s, and RC = 6°/s. Applying equation (1) for JF yields a unilateral weakness (UW) value of 39%, exceeding the normal cutoff of 25%. In contrast, using equation (6) for SEF results in a linear paresis value of 56%, surpassing the corresponding 40% cutoff. The vHIT result in B from the same patient aligns with the caloric test findings, demonstrating right-sided peripheral vestibular hypofunction and confirming the clinical diagnosis. Abbreviations: Jongkees’ formula (JF), unilateral weakness (UW), left (L), right (R), hot (h), cold (c), slow-phase velocity (SPV).

To illustrate the differences between applying JF and SEF to real-world data, Figure 4 presents a typical caloric test report from a patient with a unilateral vestibular deficit on the right side. The reports were generated using Interacoustics VNG (A) and Interacoustics EyeSeeCam vHIT (B) systems (Interacoustics, Middelfart, Denmark). When the slow-phase velocity responses—23°/s, 24°/s, 9°/s, and 6°/s for LH, LC, RH, and RC caloric stimulations, respectively—are entered into equation (1), the resulting JF value is 39%, close to the point where JF underestimation is maximal. In contrast, applying equation (6) for SEF yields a value of 56%, which more intuitively represents the extent to which the affected right ear’s responses are weaker than those of the left ear.

**Figure 5.**
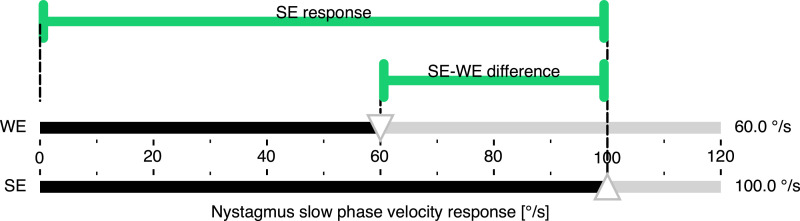
Calorigram of a hypothetical unilateral vestibular disorder illustrating the SEF calculation. The nystagmus response of the affected WE (top) is set to 60% of the SE (bottom). As shown in Figure 3, this corresponds to the clinical threshold asymmetry of 40% when calculated with the SEF. The magnitudes representing the SE-WE difference and the SE response, corresponding to the numerator and denominator of the SEF, respectively, are shown using the same color coding as for SEF in Figure 3. Abbreviations: strong ear formula (SEF), weaker ear (WE), stronger ear (SE).

JF is a bilateral test formula used for a unilateral caloric test. It uses contributions from both sides for the reference value. Including the WE value, which varies considerably among patients, in the denominator in addition to the SE results in a nonlinear characteristic (see Figure 3) that is not intuitive to the clinician.^3^ Our analysis confirms the shortcomings of this approach, which Wexler has shown already in 1994.^12^ The measure of unilateral weakness obtained by applying JF may be misleading because the true degree of vestibular hypofunction is greater than the percentage given by JF. Because in the caloric test it is not possible to determine an absolute reference value, the stronger ear response should be used as a reference instead, as suggested by Wexler. This approach can more intuitively be understood as a measure of unilateral weakness, as illustrated in Figure 5.

### Artificially inflated results

Despite its linearizing effect, the SEF equation (6) still has two disadvantages. The sign indicating the side of the loss is not preserved, leading to a distorted normal range and difficulties in longitudinal analysis. More importantly, low SE values lead to artificially inflated results.^10^ In a bilateral vestibulopathy with low SE responses, both the linear SEF and the nonlinear JF become very sensitive to small changes in WE and quickly reach inflated results. In such an extreme case, even a clinically irrelevant side-to-side difference in the range of the intra-organ variation or the smallest worthwhile change^1^ may become artificially inflated.^11,12^

With the caloric test showing a convection-dependent response, the interpretation of the metrics in bilateral vestibulopathy is left to the clinician. To avoid artificially inflated results in these cases, unilateral weakness is not recommended to be calculated any longer when the sum of both nystagmus responses per ear falls below a safe criterion of 6°/s.^23^

### Clinical relevance and normative ranges

**Figure 6.**
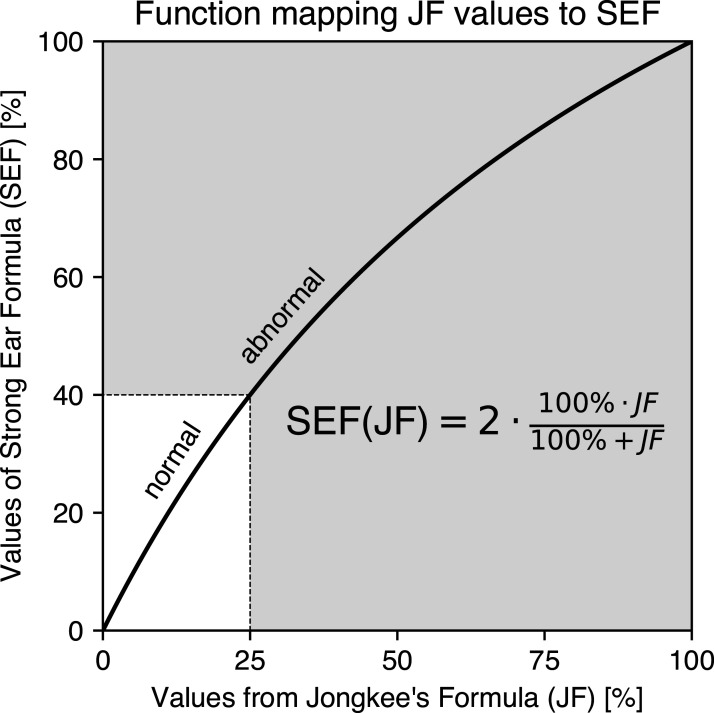
Function for converting JF values directly into their SEF equivalents. When a JF value (in %) is input into the SEF(JF) function, the output is the corresponding SEF value (in %). For instance, the cutoff value of 25%—which separates normal (white) from abnormal (gray) values in the JF co-domain—is mapped along the dotted lines to its equivalent cutoff value of 40% in the SEF co-domain. This example highlights the underestimation of unilateral weakness in the JF co-domain, where an asymmetry index of 25% actually corresponds to a weakness of 40%. The nonlinearity of the SEF (JF) function captures this underestimation across the entire range of JF values (0%–100%). The functions derivation is provided in the Appendix, along with a link to a web application implementing it.

When making a binary diagnostic decision based on an instrument-derived metric, a clinician’s primary concern is whether the metric falls within or outside its reference range, typically determined by cutoff values. Following the approach proposed by Wexler,^12^ Figure 3 illustrates how the JF cutoff value of 25% can be mapped graphically to its SEF counterpart of 40%. Instead of relying on this intuitive graphical mapping, a function SEF(JF) can be derived from Equations (e) and (f) to directly convert JF values to their SEF equivalents. Figure 6 visualizes this mapping function, with its mathematical derivation provided in the Appendix.

The classification of an SEF value as normal or abnormal is thus based on a comparison with the mapped 40% cutoff value, just as the classification in the JF co-domain relies on the 25% cutoff. This interchangeability allows clinicians to use SEF and JF seamlessly without altering diagnostic outcomes. Consequently, existing reference databases do not need to be recalculated—there is no need to re-enter four peak eye velocity measurements per sample to compute SEF reference ranges. Instead, clinicians can simply apply the conversion formula from Figure 6 and compare the result with the mapped cutoff.

Given the mathematical interchangeability of JF and SEF calculations, one might question the necessity of SEF when JF has been widely used for decades. The answer lies in JF’s inherent underestimation of vestibular paresis. For instance, a true paresis of 40% is reported as only 25% when using JF, as demonstrated in Figures 3 and 5. The SEF value of 40% accurately quantifies the reduction in oculomotor response, whereas the JF value of 25% can be misleading in this context. Therefore, we recommend that diagnostic device manufacturers implement SEF to provide clinicians with a more intuitive and realistic representation of caloric test metrics.

## Improved asymmetry calculation in vHIT

One might be tempted to apply the SEF from equation (6) to the vHIT as well,^24^ since it would linearize the result and quantify the expected degree of response deficit in the weaker ear relative to the stronger ear. However, this is only an important expectation for the caloric test, which lacks an absolute reference, but not for the vHIT, which uses head velocity as a reference for each impulse. To improve the calculation of asymmetry in the vHIT, we suggest using the ideal VOR gain of 1 as the reference value in the denominator, rather than either the sum norm or the maximum norm. This also eliminates having to decide whether the vHIT is bilateral or unilateral.

**Figure 7.**
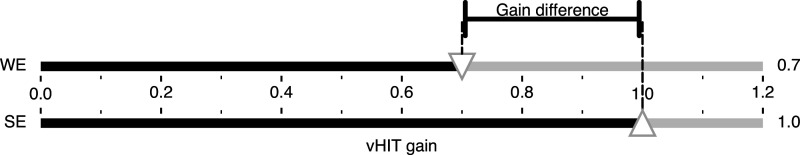
vHIT gains and their difference resulting from head rotations ipsiversive (top) and contraversive (bottom) to a hypothetical unilateral vestibular deficit. The gain of the healthy ear is set to the ideal unity gain and the gain of the affected ear is set to 0.7, which is the clinical threshold for a vestibular deficit. Equations (7) and (5) yield the same value of 30% for IGF and SEF, respectively, but equation (3) yields an underestimated value of JF = 18%.

In light of these considerations, we can formulate an ideal gain formula (IGF) for vHIT using ideal unity gain as the reference value. Since the denominator disappears at a value of 1, the IGF calculation is reduced to just the side-to-side difference as an intuitive measure of asymmetry that is easy for clinicians to calculate (Figure 7):

(7) IGF=(L−R)/1=(L−R)×100%

**Figure 8.**
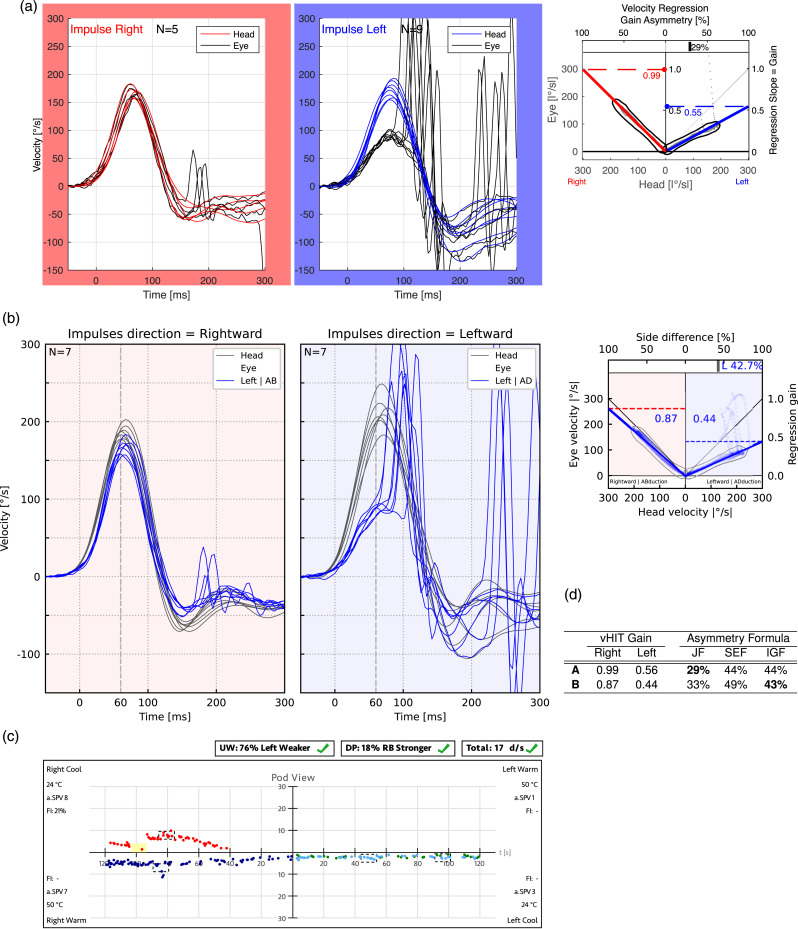
vHIT test reports (a) and (b) and table with corresponding results (d) from a patient with a unilateral vestibular deficit on the left side, confirmed by caloric test (c). Eye and head velocity traces are shown for head impulses to the right (right frames) and left (blue frames). Panel (a) displays data from a monocular vHIT system, which reports the JF value as a measure of asymmetry, while panel (b) presents results from a newer binocular vHIT system, which reports the IGF value. Regression slopes on the right indicate VOR gains, which are listed in Table (d) alongside asymmetry values calculated using JF, SEF, and IGF. Bold values highlight the asymmetry measure reported by each vHIT system. Caloric irrigation (c) confirmed left-sided areflexia in this patient. Abbreviations: Jongkees’ formula (JF), ideal gain formula (IGF), unilateral weakness (UW), video head impulse test (vHIT), vestibulo-ocular reflex (VOR).

To illustrate the differences between applying JF, IGF, and SEF to real-world vHIT data, Figure 8 presents typical vHIT test results of a patient with a unilateral vestibular deficit on the left side. The reports were generated using an established monocular Interacoustics EyeSeeCam vHIT system (A) reporting the JF value and a newer binocular EyeSeeCam Sci 2 vHIT system (B) already reporting the IGF value (EyeSeeTec, Munich, Germany). When the gain values of 0.55 for the weaker left ear and 0.99 for the stronger right ear are entered into equation (3), the resulting JF asymmetry value is 29%. In contrast, applying equation (6) for SEF and equation (7) for IGF yields asymmetry values of 44% and 43%, respectively—values that more intuitively reflect the extent to which the affected left ear’s gain is weaker than that of the right ear.

Clinicians relying on reference ranges for JF in diagnostic decision-making may struggle to find established cutoff values for vHIT. A cutoff of 5.6% has been reported for head impulse testing using the gold-standard—but invasive—search coil method.^5,19^ However, for vHIT, only a few normative data studies are available,^5,7,25^ highlighting the need to establish normative values for vHIT gain asymmetry based on IGF and to initiate a consensus process on this matter.

### Preventing artificially inflated results

**Figure 9.**
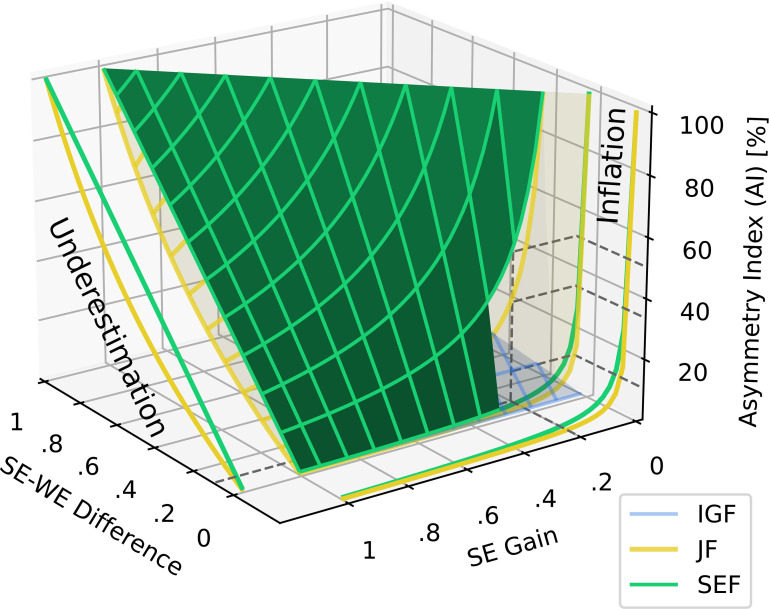
Asymmetry index calculations using the SEF, IGF, and nonlinear JF methods across the plane of all possible SE gain and SE-WE difference values. Exploded views of JF and SEF illustrate the effects of underestimation along the SE-WE axis and, more importantly, inflation along the SE axis. The “Underestimation” view provides insights similar to the top part of Figure 3, specifically the exploded JF and SEF graphs. To facilitate comparison, the color scheme from Figure 3 is retained. The JF and IGF estimates of unilateral weakness are consistently lower than SEF, causing the SEF surface to always exceed and conceal the other surfaces. The exploded “Inflation” view illustrates how inflation occurs as SE approaches zero when the SE-WE difference is at its minimum value of 0.01, leading to significant asymmetry index variations of JF and SEF. To better visualize the inflation of JF and SEF—but not IGF—in the low SE and SE-WE range, the SEF surface is partially incised. This reveals that at SE = 0.2 and SE-WE = 0.1, JF and SEF produce inflated asymmetry values of 33% and 50%, respectively, whereas IGF remains stable at 10%. These example values are indicated by dashed gray lines. Abbreviations: Jongkees’ formula (JF), weaker ear (WE), stronger ear (SE), strong ear formula (SEF), ideal gain formula (IGF), asymmetry index (AI).

By varying one VOR gain from 1 to 0 while keeping the other constant in equation (7), it becomes evident that the sum norm’s nonlinearity is eliminated and that the asymmetry is not underestimated. Similar to Figure 3, this is visualized in Figure 9 along the “Underestimation” label.

In addition, Figure 9 also illustrates how both JF and SEF produce inflated vHIT asymmetries in the low SE and SE-WE range, similar to the caloric test asymmetries discussed in Section 2.4. Using the ideal gain formula prevents these artificial inflations because, unlike JF or SEF, it does not rely on a denominator that can be influenced by low bilateral gain values entering a sum or maximum norm. For instance, with a SE-WE difference of 0.1, vHIT gains of 0.2 and 0.1 result in a meaningful IGF of 10%, whereas JF and SEF yield inflated values of 33% and 50%, respectively, as depicted in Figure 9, with dashed lines indicating the corresponding values.

To prevent inflated results, the clinician can either use IGF or must rule out bilateral hypofunction by ensuring that the gain is not less than 0.6 on both sides.^23^ At the same time, it’s important to note that zero asymmetry does not mean that the vestibulo-ocular system is functioning properly; it only indicates symmetry.

## Conclusion

Quantifying functional asymmetry between the left and right vestibular organs relies on calculating an asymmetry index, which requires a reference value. It is crucial to distinguish between the different calculation methods depending on whether a test is unilateral, evaluating only one side at a time, or bilateral, evaluating both sides. In particular, during unilateral stimulation, there is no measurable simultaneous contribution from the contralateral side. Therefore, any side-to-side asymmetry should be quantified without including the contributions from both sides in the reference value.

Since 1962, caloric test reports have adopted Jongkees’ bilateral formula to assess relative unilateral weakness^3^, with the result that the calculated asymmetry can be misleading. First, because it does not directly represent the loss of vestibular function in the weaker ear compared to the stronger ear, as clinicians may assume. Due to a nonlinear characteristic that always underestimates the weakness, a change in the vestibulo-ocular reflex function produces a disproportionate change in unilateral weakness. Second, it calculates the asymmetry not from an independent physiological operating point or reference, but from a floating “symmetry point” that splits the difference between the two sides into two parts, with the average response in the middle.

To overcome these limitations of Jongkees’ formula, Wexler in 1994^12^ introduced a linear paresis calculation based on the maximum norm, using only the stronger ear’s response as the unilateral reference in the denominator. For medical device manufacturers, implementing this linear paresis alongside Jongkees’ formula would require only a software update, with no need to establish new normative databases. Since values and cut-offs from one formula can be directly mapped to the other, this addition would seamlessly integrate into existing diagnostic frameworks, while simultaneously providing clinicians with a more intuitive way to interpret results. However, even with these improvements, a third limitation remains: the results can still be artificially inflated and highly sensitive to small variations in the weaker ear’s response, particularly when both ears are affected.

Unlike the caloric test, the vHIT calculates gain using an absolute reference. While some laboratories and instrument manufacturers have adopted Jongkees’ formula for calculating asymmetry, we advocate for the future use of the ideal gain formula instead or in addition. This approach simplifies calculations by reducing asymmetry assessment to the side-to-side difference alone, without compromising diagnostic accuracy. Similar to the implementation of the strong ear formula for caloric testing, adopting the ideal gain formula in vHIT would require only a software update. However, a consensus process is needed to establish normative values for this gain asymmetry measure. Interestingly, relying on the side-to-side difference takes us back to the first attempt by Fitzgerald and Hallpike^2^ in 1942 to provide an assessment of unilateral hypofunction using the simple difference between vestibulo-ocular responses.

## Appendix

### Derivation of Jongkees’ formula

For a more rigorous mathematical derivation that delves into the question of what question JF actually answers, let’s denote the average A = (SE + WE) / 2 and express SE = 2A – WE and WE = 2A – SE. Then we can use these substitutions in JF (using one equation each time):

JF=(SE−WE)/(SE+WE)

=(2A−WE−WE)/(2A−WE+WE)

=(2A−2WE)/(2A)

(8)
=(A−WE)/A

This formulation of JF answers the question:

“how far is the weaker side from the average (relative to the average).”

JF=(SE−WE)/(SE+WE)

=(SE−2A+SE)/(SE+2A−SE)

=(2SE−2A)/(2A)

(9)
=(SE−A)/A

This formulation of JF answers the question:

“how far is the stronger side from the average (relative to the average).”

### Derivation of mapping functions for Jongkees’ formula

We start by recalling the Jongkees’ formula expressed in terms of q = WE/SE as in equation (5).

JF=(1−q)/(1+q)×100%

Dividing both sides by 100% and rearranging yields

q=(1−JF/(100%))/(1+JF/(100%))

Next, we evaluate 1-q as

1−q=(2×JF/(100%))/(1+JF/(100%))

and substitute this expression into the equation (6) which expresses SEF as a function of q

SEF=(1−q)×100%

to obtain the relation between SEF and JF (both in per cent):

(10)
SEF(JF)=2×100%×JF/(100%+JF)

To facilitate the conversion, we created a web application allowing the user to enter calculated JF value and obtain SEF and the defining factor q = WE/SE, as well as to visualize the differences: https://ondrejtichacek.github.io/asymmetry_conundrum/

### Average norm

Hypothetically, another function resembling JF could be defined using the average of the total response as the norm in the denominator^1,8,22^:

(11)
$${\text{JF}}^{\prime }=\left(\text{SE}\;–\;\text{WE}\right)/\left(\left(\text{SE}+\text{WE}\right)/2\right)\;x\;100\;\left(\%\right)$$

The calculated result would then reflect the relative distance between WE and SE standardized to the average value. Compared to the sum norm in equation (2), this equation doubles the JF percentage result. Using the values from the previous example, the result would thus be doubled from 33% to 66%, reflecting the “distance” between SE and WE standardized to their average response value. In the extreme case of a complete unilateral loss with a WE response of 0, a clinically meaningless asymmetry of 200% would result. To our knowledge, this equation is not used in the vestibular literature.
